# A Geospatial Analysis of the Lung Cancer Burden in Philadelphia, Using Pennsylvania Cancer Registry Data from 2008–2017

**DOI:** 10.3390/ijerph22030455

**Published:** 2025-03-20

**Authors:** Russell K. McIntire, Katherine Senter, Christine Shusted, Rickisa Yearwood, Julie Barta, Scott W. Keith, Charnita Zeigler-Johnson

**Affiliations:** 1 College of Health, Lehigh University, Bethlehem, PA 18015, USA; 2 College of Population Health, Thomas Jefferson University, Philadelphia, PA 19107, USA; katherine.senter@jefferson.edu; 3 Division of Pulmonary and Critical Care Medicine, Sidney Kimmel Medical College, Thomas Jefferson University, Philadelphia, PA 19107, USA; christine.shusted@jefferson.edu (C.S.); julie.barta@jefferson.edu (J.B.); 4 Fox Chase Cancer Center, Temple University Health System, Philadelphia, PA 19111, USA; rickisa.yearwood@fccc.edu (R.Y.); charnita.zeigler-johnson@fccc.edu (C.Z.-J.); 5 Division of Biostatistics and Bioinformatics, Sidney Kimmel Medical College, Thomas Jefferson University, Philadelphia, PA 19107, USA

**Keywords:** lung cancer, geographic information systems, Philadelphia, cancer cluster

## Abstract

(1) Background: Lung cancer is the deadliest and second most prevalent cancer in Pennsylvania (PA), and African American patients are disproportionately affected. Lung cancer morbidity and mortality in Philadelphia County are among the highest in PA. Geographic information systems (GIS) are useful to explore geospatial variations in the cancer burden and risk factors. Therefore, we used GIS to analyze the lung cancer burden in Philadelphia to assess which areas of the city have the highest morbidity and mortality, identify potential clusters, and determine which census tract-level characteristics were associated with higher tract-level cancer burden. (2) Methods: Using secondary data from the Pennsylvania Cancer Registry, age-adjusted standardized incidence and mortality ratios (SIR and SMR) were calculated by census tract, and choropleth maps were created to visualize geographic variations in the disease burden. Two geostatistical methods were used to determine the presence of lung cancer clusters. Multivariable regression analyses were performed to identify which census-tract level characteristics correlated with a higher lung cancer burden. (3) Results: Three distinct geographical lung cancer clusters were identified. After controlling for demographics and other covariates, adult smoking prevalence, prevalence of chronic obstructive pulmonary disease, and percentage of residential addresses vacant were positively associated with higher lung cancer SIR and SMR. (4) Conclusions: Our findings may inform cancer control efforts within the region and guide future municipal-level GIS analyses of the lung cancer burden.

## 1. Introduction

### 1.1. Lung Cancer Morbidity and Mortality: A Global and Local Public Health Issue

Lung cancer is the leading cause of cancer-related death for both men and women in developed countries [1,2]. In 2024, an estimated 234,580 new lung cancer cases will be diagnosed, and 125,070 Americans will die from lung cancer [3]. Recently, lung cancer incidence has begun to decline in men across the United States; however, lung cancer rates have remained stagnant for women, indicating a persistent public health threat despite the overall decline of tobacco use [1]. Despite the nationwide decline, lung cancer mortality exceeds the national average in 21 states, including Pennsylvania [4].

Pennsylvania’s lung cancer mortality has decreased from 54.5 per 100,000 to 32.2 in 2022 [5]. As with the national trend, mortality amongst men has decreased but has lagged for women. Cancer of the lung and bronchus continues to be the most prevalent and the deadliest invasive cancer in Pennsylvania, costing approximately USD 73,800 for initial care and upwards of USD 124,000 for end-of-life care per patient in the U.S. [6]. Moreover, clear racial disparities in the lung cancer burden affect the state as well, with an incidence of 69.6 per 100,000 for African American Pennsylvanians compared to only 60.4 per 100,000 cases for White Pennsylvanians in 2023 and higher mortality in African American (38.5 per 100,000) compared to White (33.9 per 100,000) Pennsylvanians from 2016 to 2020 [7,8].

From 2012 to 2016, Philadelphia County had among the highest lung cancer incidence and mortality rates in the state compared to other counties—and significantly higher rates than the U.S. overall [9]. Further, a recent cluster analysis of lung cancer in the Greater Philadelphia area identified zip codes within Philadelphia County as having significantly higher lung cancer incidence rates than zip codes in the surrounding counties [10,11]. Further analysis at a more local level may illuminate how lung cancer is distributed in individual Philadelphia neighborhoods and thereby inform neighborhood-level cancer control efforts [12,13,14,15].

### 1.2. Emerging Emphasis on Social and Neighborhood Determinants of Cancer

Despite decades of research that have identified several well-known risk factors for lung cancer, such as smoking, indoor and outdoor air pollution, and occupational and environmental carcinogens [1,16], translating this knowledge into effective mitigation strategies across diverse populations continues to present a public health challenge. Studies have investigated the social and neighborhood factors associated with cancer morbidity and mortality to illuminate how to more effectively target interventions [17,18,19,20]. One analysis of California Cancer Registry data from 2000 to 2013 determined that the stage of diagnosis, neighborhood socioeconomic status, and marital status were most strongly associated with racial and ethnic disparities in cancer survival [17]. Racial and ethnic disparities in lung cancer mortality were more pronounced in women compared to men [17]. Another study of California Cancer Registry data found that despite an overall decrease in lung cancer incidence, the reduction in female lung cancer cases was most significant in areas with higher neighborhood socioeconomic status [19]. Thus, understanding which factors impact lung cancer morbidity and mortality at the neighborhood level may inform why improvements in lung cancer outcomes are not equally distributed among neighborhoods.

In a retrospective analysis of lung cancer incidence using electronic medical record (EMR) data from an Ohio healthcare system, Adie et al. created a “deprivation index” to approximate neighborhood living conditions using 15 census-tract level socioeconomic variables [21]. The study concluded that even when controlling for key demographic characteristics and smoking history, increases in the neighborhood deprivation score were significantly associated with a higher hazard ratio for the presence of a lung cancer diagnosis. The authors conclude that consideration of neighborhood socioeconomic characteristics may inform health promotion and screening efforts and that their findings warrant further study of the mechanism of how neighborhood-level deprivation affects lung cancer risk [21].

### 1.3. Geographic Information Systems and the Assessment of Cancer Burden

One means of studying neighborhood factors and their association with cancer diagnoses is to geographically analyze the distribution of cancer in an area through geographic information systems (GIS), which allow users to assess the burden of disease visually and statistically in a designated geographic context [22,23]. For example, researchers in Shandong Province, China, assessed urban–rural differences in the geospatial distribution of lung cancer and identified clusters of lung cancer mortality within the province [24,25].

At the municipal level, an analysis of the geospatial distribution of breast, cervical, and colorectal cancer incidence in Baltimore found that incidence and neighborhood-level factors varied by cancer type across neighborhoods [15]. In Philadelphia, McIntire et al. created prostate [14] and breast cancer [13] composite scores, composed of morbidity, mortality, and aggressiveness, to identify census tracts with a high cancer burden and thereby best prioritize cancer control efforts. However, to our knowledge, a parallel geospatial analysis of the lung cancer burden restricted to a single city has not yet been published, despite clear evidence that known lung cancer risk factors such as air pollution and environmental carcinogens may influence the geospatial distribution of disease, in addition to neighborhood-level socioeconomic status and behavioral patterns [15,24].

Prior studies provided a precedent for analysis of neighborhood characteristics and lung cancer burden in state cancer registry data but mainly comprised of statewide multivariable analyses [19] with limited application in the context of local lung cancer prevention, screening, and treatment strategies. Given the high rates of lung cancer morbidity and mortality in Philadelphia, understanding the spatial distribution of lung cancer in Philadelphia is essential to best target limited healthcare resources. A local-level geospatial analysis of lung cancer burden might also aptly inform additional analyses at NCI-designated cancer centers and their catchment areas around the country, especially given the limited number of prior geospatial cancer analyses conducted at the neighborhood scale or using residential locations [22].

Therefore, we hypothesized that by conducting a geospatial analysis, we would be able to (1) determine the spatial distribution of lung cancer burden in Philadelphia, including identification of potential lung cancer clusters, and (2) identify potential neighborhood correlates associated with the lung cancer burden in Philadelphia, to better guide cancer control efforts in the region and to inform municipal geospatial lung cancer control assessments.

The purpose of this study was to spatially characterize lung cancer morbidity and mortality in Philadelphia, to identify any lung cancer clusters, and to assess the relationship between census tract-level demographic, socioeconomic, morbidity, and behavioral characteristics with lung cancer morbidity and mortality. We sought to inform cancer control efforts in the region and further the theoretical basis for geographically prioritizing area-level interventions to reduce the burden of lung cancer.

## 2. Materials and Methods

### 2.1. Data Sources

We conducted a secondary analysis of ten years of Pennsylvania Cancer Registry data from 2008 to 2017. The registry data included residential address, age, race, marital status, and cancer history of all reported Pennsylvania lung cancer cases. For the present study, only cases from Philadelphia County (*n* = 12,013) were included. The data was cleaned, then addresses were geocoded using Environmental Research Systems Research Institute’s (ESRI) ArcMap 10.8.1 software, building an address locator based on Philadelphia Streets Department street centerlines data (2021) to accurately match cases to their geographic coordinates on the base map of Philadelphia. For addresses that did not automatically match with our locator, we manually verified the address and corrected any typographical errors before attempting rematch to ultimately achieve a 98% match rate (*n* = 11,767).

The matched lung cancer cases were aggregated by census tract using the 2019 TigerLine shapefiles from the U.S. Census Bureau [26]. Eleven census tracts were excluded and marked as nonresidential, defined as tracts with a 2010 Census population of less than 300, to ensure that any tract identified as an area of concern would include sufficient inhabitants to engage in potential interventions [13,14]. After excluding nonresidential areas, 373 census tracts were included in the analysis. A subset of the data was created for cases that were reported to have died due to cancer of the lung and bronchus per applicable International Classification of Diseases (ICD)-10 codes, *n* = 6978.

To measure census tract-level characteristics, additional data were obtained from Policy Map and the Centers for Disease Control and Prevention (CDC) 500 Cities Project, which provided geographically linked data from government data sources for socioeconomic and health-related indicators [27,28]. To account for census tract-level characteristics, we used U.S. Census data (2010) and American Communities Survey data (2010–2014) to measure median household income, percentage of population African American, percentage of population White, percentage of population Asian, percentage of population Hispanic, percentage lacking health insurance, homeownership rate, and percentage of residential addresses vacant. Additional census tract-level health indicators were obtained from the 2017 CDC 500 Cities data, including adult smoking prevalence; prevalence of adults diagnosed with chronic obstructive pulmonary disease (COPD), emphysema, or chronic bronchitis; percentage of adults reporting a routine physical exam in the past year; and percentage of adults reporting 14 or more poor physical health days during the past 30 days [28]. Census-tract level health indicators from Policy Map’s Behavioral Risk Factor Surveillance System (BRFSS)/U.S. Census 2018 dataset included the percentage of adults with obesity and the percentage of adults achieving the recommended level of physical activity.

### 2.2. Statistical Analysis

Using indirect age-adjustment methodology, standardized incidence ratios (SIR) and standardized mortality ratios (SMR) were calculated by census tract using the 2010 Census population counts for Philadelphia census tracts and the Surveillance Epidemiology and End Results (SEER) U.S. incidence rates (2013–2017) and U.S. mortality rates (2014–2018) for cancer of the lung and bronchus [29,30]. Lung cancer age-adjusted SIR and SMR (referred to as SIR and SMR, respectively, for the remainder of this analysis) by Philadelphia census tract were visualized on graduated choropleth maps to assess areas of higher and lower than expected lung cancer burden after the indirect age-adjustment method. As part of the city’s plan for rejuvenation, Philadelphia has been divided into 18 planning districts. These districts contain many neighborhoods, numerous census tracts, and cross zip codes. Planning districts will be used for descriptive purposes in this paper to orient the reader to particular areas of focus.

For the cluster analysis, lung cancer SIR and SMR “hot spots and cold spots” were identified using Getis Ord Gi* statistical calculations within ArcMap to detect local groupings of census tracts with non-random spatial distribution of high or low values, i.e., instances where neighboring tracts demonstrated similarly high or low disease burdens with statistical significance at the *p* ≤ 0.10, *p* ≤ 0.05, and *p* ≤ 0.01 levels [31]. Cluster and outlier maps of SIR and SMR were created using Local Indicators of Spatial Association (LISA), which incorporate the Local Moran’s I Statistic values of all census tracts within the study area to determine groupings of high- and low-value census tracts and to compare the value of each tract to the global (non-neighboring) tracts, to thereby identify both clusters and outliers [32]. Local Moran’s I and Getis Ord Gi* calculations were used to identify census tracts with statistically significant high lung cancer burdens per both methods. Census tracts were classified as part of lung cancer clusters if they were designated as both “hot spots” per Getis Ord Gi* z-scores and “high–high” per Local Moran’s I calculation to triangulate our measurement of spatial association [15,32,33]. Cluster maps for SIR and SMR were created to display census tracts identified as areas of concern that had an overlap between the two clustering techniques.

Lastly, multivariable linear regression models were built using IBM SPSS Statistics 26 [34] to determine which census tract-level sociodemographic characteristics were associated with census tract lung cancer SIR and SMR. Univariate descriptive statistics were calculated to assess the mean and standard deviation of SIR, SMR, and independent variables for each of the 373 census tracts. Bivariate Pearson correlations were calculated for SIR and SMR with each independent variable. Multivariable linear regression models were then used to estimate regression coefficients (β) and confidence intervals (CI) to identify which census tract-level characteristics were associated with higher lung cancer SIR and SMR at the α = 0.05 level.

This analysis was one of several to be conducted under a Thomas Jefferson University Institutional Review Board-approved protocol, which aimed to analyze Pennsylvania Cancer Registry lung cancer data.

## 3. Results

### 3.1. Geographic Distribution of SIR and SMR

**Figure 1:**
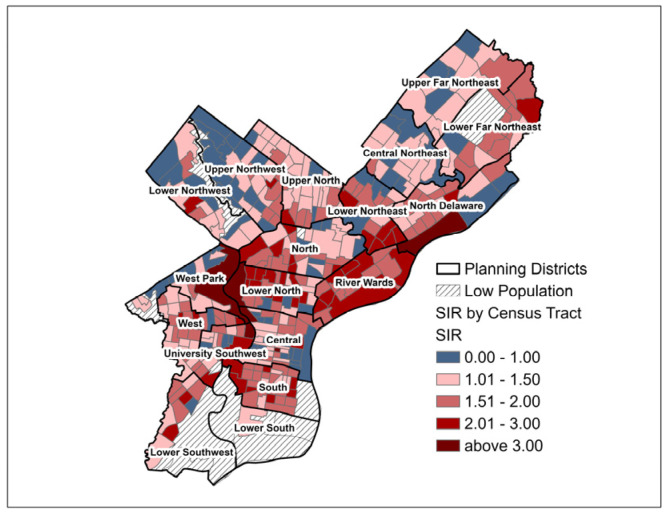
Lung Cancer Standardized Incidence Ratio (SIR) by Philadelphia, PA Census Tract for Pennsylvania Cancer Registry Data 2008 to 2017.

**Figure 2:**
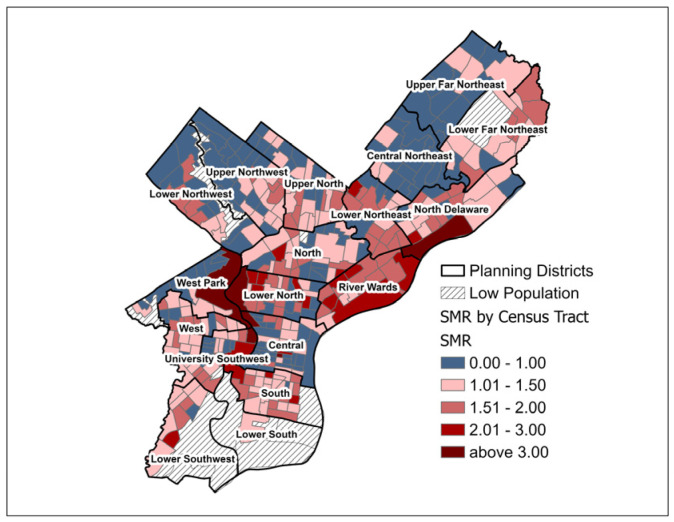
Lung Cancer Standardized Mortality Ratio (SMR) by Philadelphia, PA Census Tract for Pennsylvania Cancer Registry Data 2008 to 2017.

The mean lung cancer SIR for the 373 Philadelphia census tracts was 1.46 (SD = 0.55). The majority of census tracts (304; 81.2%) had an SIR of greater than 1.00 (i.e., higher than expected in the age-adjusted population after standardizing by national lung cancer rates). Several city planning districts, such as Lower North and River Wards, included groups of census tracts with an SIR of 1.5 to 2.0 or 2.1 to 3.0 (Figure 1). The mean SMR was 1.23 (SD = 0.54), and the SMR was greater than 1.00 for 236 out of 373 tracts. Visual comparison of the choropleth maps of SIR and SMR indicated a similar geographic distribution of higher and lower values, although the map of SMR included more census tracts with values of less than or equal to one, primarily concentrated in parts of Northwest and Northeast Philadelphia (Figure 1 and Figure 2). Upon visual inspection, the following planning districts demonstrated groupings of census tracts with the lowest SIR and SMR (less than or equal to 1.0): Lower and Upper Northwest, West Park (along the northwest border of the district), Central, and Upper Far Northeast. Although tracts with SIR and SMR > 1.5 were dispersed across many planning districts, groupings of census tracts with the highest SIR and SMR (>2.0) were apparent in the Lower North, West, University Southwest, South, and River Wards planning districts.

**Figure 3:**
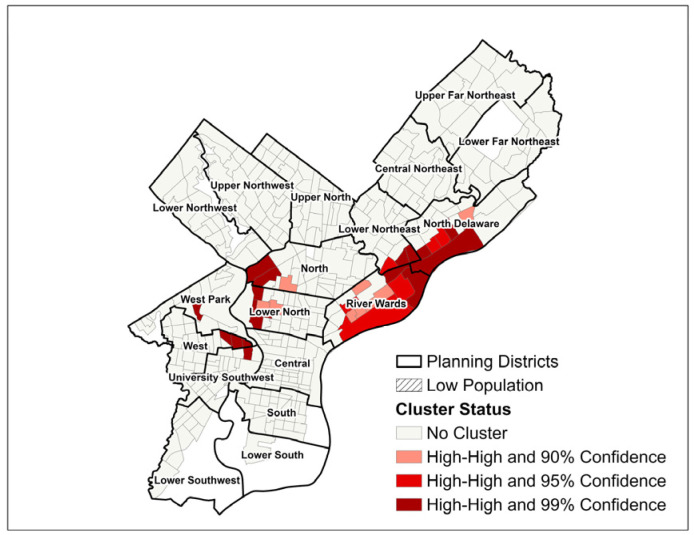
Lung Cancer Standardized Incidence Ratio (SIR) Clusters by Philadelphia, PA Census Tract for Pennsylvania Cancer Registry Data 2008 to 2017.

**Figure 4:**
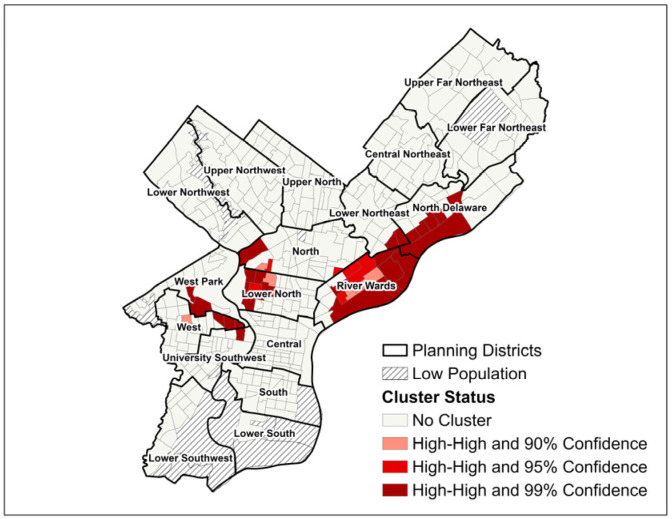
Lung Cancer Standardized Mortality Ratio (SMR) Clusters by Philadelphia, PA Census Tract for Pennsylvania Cancer Registry Data 2008 to 2017.

Fifty-five census tracts met both Moran’s I and Getis-Ord Gi* criteria to be classified as part of SIR clusters, and 40 census tracts met the criteria for SMR clusters. For both SIR and SMR, two distinct geographic clusters of high-burden census tracts were apparent: one cluster in the North/Lower North planning districts bordering Fairmount Park, and one cluster spanning parts of the River Wards, Lower Northeast, and North Delaware planning districts (Figure 3 and Figure 4). For SIR only, a third cluster was apparent in the western half of the South Philadelphia planning district.

### 3.2. Multivariable Model of Census Tract-Level Predictors of SIR and SMR

Table 1 shows the mean, standard deviation (SD), and bivariate Pearson correlations with lung cancer SIR and SMR for each tract-level characteristic. This table indicates that the following variables demonstrated a positive unadjusted association (*p* < 0.05) with both SIR and SMR: percentage of population African American, home ownership rate, percentage with 14 or more reported poor health days over the past 30 days, and percentage of residential addresses vacant. Median household income, percentage of population White, percentage of population Asian, and percentage who achieved the recommended amount of physical activity demonstrated negative correlations with lung cancer SIR and SMR.

While controlling for covariates in our multivariable regression models (Table 2), several census tract-level variables demonstrated statistically significant increases in both census tract lung cancer SIR and SMR associated with 10 percent increases in adult smoking prevalence (SIR β = 0.654; 95% CI: 0.163, 1.128; SMR β = 0.673; 95% CI: 0.183, 1.163), COPD prevalence (SIR β = 2.805; 95% CI: 0.580, 5.030; SMR β = 2.995; 95% CI: 0.734, 5.255), and residential addresses vacant (SIR β = 0.130; 95% CI: 0.050, 0.209; SMR β = 0.112; 95% CI: 0.031, 0.192). A 10 percent increase in the census-tract level population with 14 or more reported poor health days over the past 30 days was negatively associated with lung cancer SIR and SMR (SIR β = −1.918; 95% CI: −3.499, −0.337; SMR β = −2.144; 95% CI: −3.750, −0.538). Additionally, increases in SIR were associated with a 10 percent increase in census-tract level obesity (SIR β = 0.613; 95% CI: 0.012, 1.214).

## 4. Discussion

Through geospatial analysis and multivariable modeling of lung cancer SIR and SMR at the census tract level, our study geographically characterized the burden of lung cancer in Philadelphia and identified several factors associated with a higher lung cancer burden at the census tract level. Our census tract-level analyses highlighted the extent to which lung cancer morbidity and mortality in Philadelphia were higher than expected compared to national age-adjusted lung cancer rates, underlining the high lung cancer burden for Philadelphia County [9]. The mean census tract-level age-adjusted lung cancer SIR of 1.46 indicates that lung cancer incidence in the average census tract in Philadelphia from 2008 to 2017 was 46% higher than expected, based on the age distribution of people in the census tracts, after standardizing with national lung cancer rates. Similarly, the SMR of 1.23 signifies that the average census tract-level lung cancer mortality rate is 23% higher than expected. These results emphasize the ongoing need for heightened lung cancer control efforts in Philadelphia.

The identification of three distinct lung cancer SIR clusters may inform the geographic prioritization of lung cancer prevention, screening, and treatment efforts in Philadelphia. Evidence-based interventions to decrease lung cancer incidence and mortality aim to reduce smoking initiation and increase smoking cessation, as well as improve efforts to screen individuals with significant smoking history using low-dose computed tomography (LDCT) scans. [1]. Despite prior development of evidence-based lung cancer screening protocols, an earlier analysis found that significantly more adults were screened inappropriately for lung cancer than were screened according to the 2013 U.S. Preventative Services Task Force (USPSTF) guidelines [35]. Discordance between real-world uptake and implementation of lung cancer screening and that recommended by clinical practice guidelines reflects a need for comprehensive and centralized approaches to improve screening access and effectiveness [36]. Revised USPTSF guidelines from 2021 recommend annual LDCT screening for adults ages 50–80 (previously ages 55–80) with a 20 pack-year smoking history (previously 30 pack-years) who either currently smoke or quit in the past 15 years. These individuals should receive annual LDCTs until they either quit smoking for 15 years or develop a health condition that “substantially limits life expectancy” [37]. More recent analyses showed that the USPSTF 2021 guideline changes broadened lung cancer screening eligibility and reduced some racial disparity in access to screening [38]. In addition to previous studies highlighting varying screening eligibility among Philadelphia neighborhoods [39], the identification of lung cancer clusters may inform the placement of additional screening outreach, resources, and/or protocol improvements in current facilities to improve lung cancer outcomes in high-burden census tracts.

The similar geographic distribution of lung cancer incidence and mortality rates likely reflects the poor survival outcomes of lung cancer. From 2012 to 2018 in the U.S., 5-year relative survival for non-small cell lung cancer for all stages at diagnosis combined was 28%, and 5-year survival for small cell lung cancer was only 7% [40]. Therefore, it can be expected that areas with higher lung cancer incidence over a ten-year period would have similarly high lung cancer mortality, as the SIR and SMR maps and cluster maps displayed. Given that even within one city planning district, several census tracts may form a cluster, while the remaining tracts in the district do not demonstrate significant spatial autocorrelation, our analysis illustrates the benefit of using neighborhood-level units of area to identify more specific areas for public health intervention, instead of using larger units such as zip codes or planning districts.

However, several differences between lung cancer incidence and mortality in Philadelphia were identified. Most notably, a third lung cancer cluster was apparent in South Philadelphia for SIR but not for SMR. Additionally, while SIR and SMR were higher than 1.0 in the majority of census tracts, fewer tracts had an SMR of >1.0 (236) than had an SIR of >1.0 (304). This contrast between the geographic areas affected by higher-than-expected incidence compared to mortality may indicate that while lung cancer is diagnosed at high rates throughout most of Philadelphia, more localized factors may impact the rate at which residents die from lung cancer. These factors may include potential neighborhood differences in the stage at diagnosis and access to health care [17]. For example, higher uptake of lung cancer screening or more intensive follow-up of incidental lung nodules can lead to a higher proportion of early-stage compared with late-stage lung cancer diagnoses [41]. Notably, our model of SMR does not incorporate the stage at diagnosis, which is a key predictor of lung cancer mortality [40].

Many of the associated census tract-level variables identified in the multivariable models of lung cancer SIR and SMR are already well-established in the literature, including smoking prevalence and the presence of a COPD diagnosis, both positively associated [1]. The association between the percentage of residential addresses vacant and the lung cancer burden is not unexpected since neighborhoods with higher vacancies tend to have worse health outcomes [42,43]. However, this may warrant further investigation as a social determinant of lung cancer outcomes since this variable remained significant when controlling for race, sex, and income. The negative association between the lung cancer burden and the percentage of the census tract reporting 14 or more poor health days over the last 30 days was unanticipated, given that self-reported health quality of life is associated with worse cancer outcomes [44,45,46]. This discrepancy may point to a limitation of ecological analyses, such that we cannot assume that patients who reported poor health were also those who were diagnosed with lung cancer [15].

Despite African American Pennsylvanians having higher lung cancer incidence and mortality compared to White Pennsylvanians, the census tract-level percentage of African Americans was no longer significant in either model after controlling for other potential confounders, indicating that neighborhood and socioeconomic factors may drive racial disparities in cancer burden, as identified in prior research [17]. The multivariable models in this analysis accounted for only 37.9% and 28.6% of variability in lung cancer SIR and SMR, respectively; a more robust, local comparison of high and low-burden tracts may illuminate additional sources of variability. As described by McIntire et al. in their analysis of the census tract-level prostate cancer burden in Philadelphia, a dual approach of targeting cancer control efforts based on the known geographic distribution of cancer rates, while also informed by health and demographic characteristics, may be the most logical application of our findings [14].

### Strengths and Limitations

The Pennsylvania Cancer Registry includes all new cancer cases diagnosed and treated in the state, making it a comprehensive representation of Philadelphia lung cancer patients from 2008 to 2017. However, since mortality data in the Pennsylvania Cancer Registry only includes the primary cause of death, our calculations of mortality likely underreport cancer-related deaths because some lung cancer patients may have died due to cancer complications that were reported as the primary cause of death, e.g., pneumonia. Moreover, the results may not mirror the current burden of lung cancer in Pennsylvania given the ongoing changes over time of the lung cancer burden in the U.S. [19]. Similarly, while the relevance of our results is limited outside of Philadelphia, our methods are reproducible and provide a valuable framework for local public health professionals to assess the cancer burden in their respective communities to appropriately target interventions.

Our statistical modeling was limited to risk factor data that is aggregated to the census tract level. Therefore, we could not measure associations between the lung cancer burden and other known risk factors, including air quality, radon, asbestos exposure, and other environmental factors, as this data is not available at the census tract level. Additionally, associations found in the cancer burden at the census tract level do not necessarily translate to the individual level. Lastly, this study is observational, and causality cannot be definitively determined from identified associations.

## 5. Conclusions

In a geographic and statistical analysis of ten years of Pennsylvania Cancer Registry data, several city planning districts in Philadelphia County with statistically significant groupings of high lung cancer incidence and mortality (clusters) were identified. Although the geographic distribution of high and low values were similar for lung cancer SIR and SMR, an additional SIR cluster was found in South Philadelphia, suggesting that certain neighborhood factors impact lung cancer survival in Philadelphia. We also identified several census tract-level predictors that were significant for both lung cancer SIR and SMR, including adult smoking, COPD prevalence, and percent residential addresses vacant. These findings may help inform cancer control efforts in the area, such as the implementation or augmentation of lung cancer screening and prevention services in the geographic areas identified as clusters.

## Figures and Tables

**Table 1:** Univariate Summary of Philadelphia Census Tracts and Bivariate Analyses Between Lung Cancer Standardized Incidence and Mortality Ratios (SIR and SMR) and Census Tract-Level Characteristics in Philadelphia, PA (*n* = 373).

Census Tract Characteristic	Mean (SD)	Pearson Correlation (SIR)	*p*-Value (SIR)	Pearson Correlation (SMR)	*p*-Value (SMR)
Lung cancer SIR ^a^	1.46 (0.55)	N/A	N/A	N/A	N/A
Lung cancer SMR ^a^	1.23 (0.54)	N/A	N/A	N/A	N/A
Median household income ($) ^b^	41,030.73 (20,864.95)	−0.325	<0.001 *	−0.335	<0.001 *
Black or African American (%) ^b^	44.44 (35.61)	0.177	0.001 *	0.176	0.001 *
White (%) ^b^	41.17 (33.00)	−0.149	0.004 *	−0.165	0.003 *
Asian (%) ^b^	6.01 (7.78)	−0.167	0.001 *	−0.153	0.033 *
Hispanic (%) ^b^	10.92 (16.34)	−0.021	0.688	0.015	0.769
Male (%) ^b^	47.16 (4.08)	−0.001	0.984	0.007	0.896
Homeownership (%) ^b^	52.71 (18.74)	0.249	<0.001 *	0.244	<0.001 *
Uninsured (%) ^c^	13.54 (6.17)	−0.023	0.662	−0.032	0.544
Adult smoking prevalence (%) ^d^	23.64 (6.22)	0.457	<0.001 *	0.452	<0.001 *
Diagnosed with COPD (%) ^d^	7.24 (2.29)	0.341	<0.001 *	0.313	<0.001 *
Obesity (%) ^e^	34.09 (5.55)	0.198	<0.001 *	0.186	<0.001 *
Achieved recommended physical activity (%) ^e^	20.58 (2.37)	−0.121	0.019 *	−0.105	0.042 *
Routine exam in past year (%) ^d^	73.85 (4.89)	0.008	0.882	−0.011	0.837
Reported 14+ poor health days (%) ^d^	14.92 (4.37)	0.321	<0.001 *	0.310	<0.001 *
Residential addresses vacant (%) ^c^	13.37 (7.49)	0.323	<0.001 *	0.305	<0.001 *

* Correlation has *p* < 0.05 (2-tailed); ^a^ Pennsylvania Cancer Registry, 2008–2017; ^b^ U.S. Census Bureau, 2010 Census via PolicyMap; ^c^ U.S. Census Bureau, ACS 2010–2014 via PolicyMap; ^d^ CDC 500 Cities: BRFSS 2017 and 2010 Census; ^e^ Policy Map: BRFSS 2018 and 2010 Census.

**Table 2:** Results from Multivariable Linear Regressions of Lung Cancer Standardized Incidence and Mortality Ratios (SIR and SMR) on Census Tract-Level Characteristics in Philadelphia, PA (*n* = 373).

Census Tract Characteristic	SIR β Coefficient (95% CI)	t-Statistic (SIR)	*p*-Value (SIR)	SMR β Coefficient (95% CI)	t-Statistic (SMR)	*p*-Value (SMR)
Median household income	−0.014 (−0.061, 0.034)	−0.568	0.570	−0.021 (−0.069, 0.027)	−0.855	0.393
African American (%)	0.077 (−0.285, 0.439)	0.417	0.677	−0.113 (−0.481, 0.255)	−0.604	0.546
White (%)	0.086 (−0.253, 0.425)	0.500	0.617	−0.123 (−0.468, 0.221)	−0.703	0.482
Asian (%)	0.132 (−0.266, 0.530)	0.654	0.513	−0.077 (−0.482, 0.327)	−0.375	0.708
Hispanic (%)	0.041 (−0.231, 0.313)	0.296	0.767	−0.036 (−0.312, 0.240)	−0.255	0.799
Male (%)	−0.112 (−0.294, 0.069)	−1.220	0.223	−0.152 (−0.336, 0.032)	−1.620	0.106
Homeownership (%)	0.011 (−0.030, 0.052)	0.520	0.603	0.018 (−0.023, 0.060)	0.864	0.388
Uninsured (%)	0.027 (−0.079, 0.133)	0.500	0.618	−0.002 (−0.110, 0.106)	−0.035	0.972
Adult smoking prevalence (%)	0.645 (0.163, 1.128)	2.632	0.009	0.673 (0.183, 1.163)	2.703	0.007
Diagnosed with COPD (%)	2.805 (0.580, 5.030)	2.479	0.014	2.995 (0.734, 5.255)	2.605	0.010
Obesity (%)	0.613 (0.012, 1.214)	2.006	0.046	0.499 (−0.112, 1.109)	1.606	0.109
Achieved recommended physical activity (%)	0.450 (−0.400, 1.299)	1.041	0.299	0.530 (−0.333, 1.393)	1.208	0.228
Routine exam in past year (%)	−0.603 (−1.271, 0.064)	−1.777	0.076	−0.525 (−1.203, 0.154)	−1.521	0.129
Reported 14+ poor health days (%)	−1.918 (−3.499, −0.337)	−2.386	0.018	−2.144 (−3.750, −0.538)	−2.625	0.009
Residential addresses vacant (%)	0.130 (0.050, 0.209)	3.206	0.001	0.112 (0.031, 0.192)	2.716	0.007

Note: Median household income was scaled to reflect a USD 10,000 change per unit in the outcome variable. All % variables were scaled to reflect a 10% change per unit in the outcome variable. SIR R^2^ = 0.379, SMR R^2^ = 0.342.

## Data Availability

The data presented in this study are available on request from the corresponding author, conditional on Thomas Jefferson University IRB and PA Cancer Registry agreements.
